# The views of patients, healthcare professionals and hospital officials on barriers to and facilitators of quality pain management in Ethiopian hospitals: A qualitative study

**DOI:** 10.1371/journal.pone.0213644

**Published:** 2019-03-14

**Authors:** Million Tesfaye Eshete, Petra I. Baeumler, Matthias Siebeck, Markos Tesfaye, Dereje Wonde, Abraham Haileamlak, Girma G. Michael, Yemane Ayele, Dominik Irnich

**Affiliations:** 1 Department of Anesthesiology, Institute of Health, Faculty of Medicine, Jimma University, Jimma, Ethiopia; 2 Centre for International Health, Ludwig-Maximilians-Universität Munich, Munich, Germany; 3 Multidisciplinary Pain Center, Department of Anaesthesiology, University Hospital Ludwig Maximilians University Munich, Munich, Germany; 4 Department of General, Visceral, Vascular and Transplantation Surgery, Hospital of the University of Munich (LMU), Munich, Germany; 5 Department of Psychiatry, St. Paul’s Hospital Millennium Medical College, Addis Ababa, Ethiopia; 6 Department of Sociology, College of Social Sciences and Humanity, Jimma, University, Ethiopia; 7 Department of Pediatrics and Child Health, Institute of Health, Faculty of Medicine, Jimma University, Jimma, Ethiopia; University of Brighton, UNITED KINGDOM

## Abstract

**Background:**

Postoperative pain remains a challenge in the developed world, but the consequences of inadequately treated postoperative pain are particularly severe in low- and middle-income countries. Since 2011, reports have drawn attention to the poor quality of postoperative pain management in Ethiopia; however, our multicenter qualitative study was the first to attempt to understand the factors that are barriers to and facilitators of quality pain managment in the country. To this aim, the study explored the perspectives of patients, healthcare professionals, and hospital officials. We expected that the results of this study would inform strategies to improve the provision of quality pain management in Ethiopia and perhaps even in other low- and middle-income countries.

**Methods:**

This study used a qualitative, descriptive approach in which nine healthcare professionals, nine patients, and six hospital officials (i.e. executives in a managerial or leadership position in administration, nursing, or education) participated in face-to-face, semi-structured interviews. Thematic data analysis was conducted, and patterns were explained with the help of a theoretical framework.

**Findings:**

The barriers identified ranged from healthcare professionals’ lack of empathy to a positive social appraisal of patients’ ability to cope with pain. They also included a lack of emphasis on pain and its management during early medical education, together with the absence of available resources. Enhancing the ability of healthcare professionals to create favorable rapport with patients and increasing the cultural competence of professionals are essential ingredients of future pain education interventions.

**Conclusions:**

Barriers to and facilitators of postoperative pain management do not exist independently but are reciprocally linked. This finding calls for holistic and inclusive interventions targeting healthcare professionals, patients, and hospital officials. The current situation is unlikely to improve if only healthcare professionals are educated about pain physiology, pharmacology, and management. Patients should also be educated, and the hospital environment should be modified to provide high-quality postoperative pain management.

## Introduction

The number of patients undergoing surgery is rising worldwide [1]. However, pain treatment after surgical procedures remains unsatisfactory [2], and up to 40% of patients experience severe pain after surgery [3]. Estimates of the proportion of patients who develop persistent postoperative pain vary from 5% up to 85% [4]. Globally, about 22% of chronic pain is related to previous surgery, but this rate can be reduced by adequate postoperative pain management [5]. Untreated postoperative pain has also been linked to extended hospital stays, atelectasis, respiratory infection, myocardial infarction [6,7], and even death [8]. In the developed world, awareness of the impact of postoperative pain has grown, but endeavors to improve its management remain challenging [3]. Patients in low- and middle-income countries (LMIC) are at greater risk of the severe consequences of untreated postoperative pain [7,9]. This is partly because pain management is not a priority in low-resource settings, where the health care systems are focused on achieving the United Nations Millennium Development Goals, such as eradicating poverty and reducing maternal and child death [10]. Other reasons why health policy in LMIC pays little or no attention to postoperative pain management, despite its importance for the prevention of disability, remain unclear. To improve the care of surgical patients in LMIC, we have to explore barriers to and facilitators of quality pain management (QPM). Although it is difficult to define and measure the quality of pain management, QPM is defined as a characteristic that encompasses the structure, process, and outcomes of care [11]. It has specific characteristics, including appropriate ongoing assessment (both before and after the administration of analgesics) and multidisciplinary, safe, efficacious, cost-effective, and culturally and developmentally appropriate care [12]. Furthermore, experts around the world currently recommend the use of multimodal regimens in many situations, although the exact ingredients can vary, depending on the patient, setting, and surgical procedure [13].

Surprisingly, at the time of writing this manuscript no qualitative data were available about the quality of postoperative pain management in Ethiopia, indicating that little or no attention is being paid to this topic. A decade ago, a nationwide study in Ethiopia reported that healthcare professionals (HCPs) believed that pain was undertreated because pain management was not standardized and analgesics, trained healthcare providers, and non-pharmacological treatment options were lacking [14]. A study from one of the hospitals participating in this study (Jimma University Medical Center) reported that the prevalence of undertreated postoperative pain was as high as 80.1%; this finding was not surprising because Ethiopia has nil morphine per capita [15,16]. A lack of strong opioids, no use of non-pharmacological pain management techniques, and poor anesthesia and surgical infrastructure are the typical characteristics of hospital settings in Ethiopia [17,18].

We decided to conduct the current study to find solutions for the above mentioned and other problems (e.g. the need to design and implement an effective intervention for QPM). The known barriers to and facilitators of postoperative pain management were mainly identified in studies conducted in the developed world and may not apply to low-resource settings or to other populations from different cultural backgrounds. Here, we present the views of patients, HCPs, and hospital officials interviewed at three hospitals in Ethiopia.

**Fig 1:**
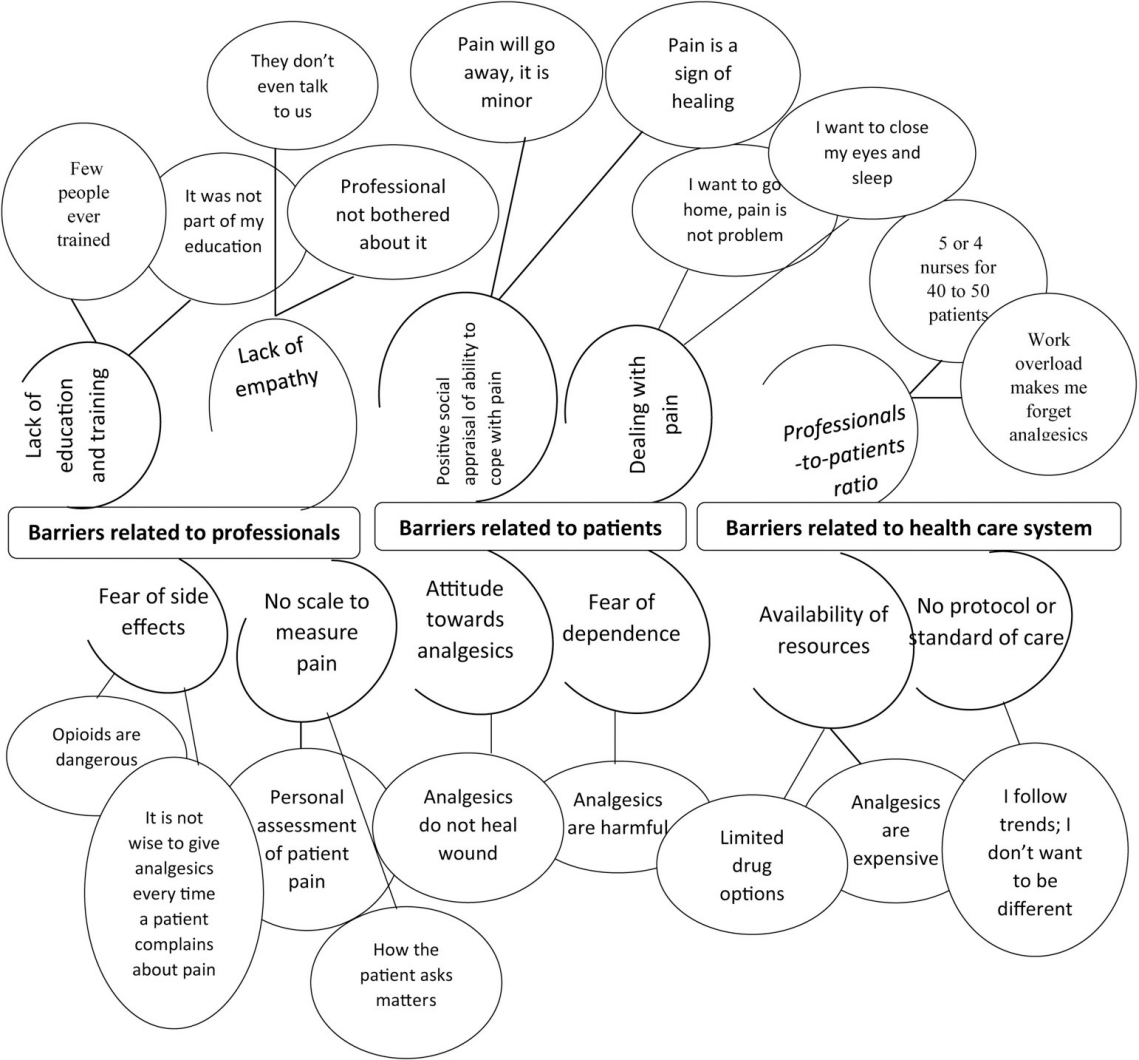
List of emergent themes that influence quality pain management.

Interview data were analyzed according to the theory of reciprocal determinism, a tenet of social cognitive theory (SCT). Reciprocal determinism has been recommended for pain management-related topics [19]. It defines behavior as a triadic, dynamic, and reciprocal interaction of personal factors, behavior, and the environment [20]. The “person” in this theory refers to the individual’s unique personality, set of experiences, personal values, cognition, thinking, etc. Environmental factors are anything outside the person, such as physical things, resources, equipment, facilities, policies, and the like. The environment also includes social factors, such as family, friends, and community [21]. Behavior is the outcome of interest, which in the current study was the “practice of postoperative pain management.” The individual, bi-directional relationship between both the personal and environmental factors and the actual behavior (current practice) is shown in Fig 1. Thus, this study used a qualitative descriptive strategy [22,23] to attempt to answer the research question of what Ethiopian patients, HCPs, and hospital officials perceive as barriers to and facilitators of quality postoperative pain management.

## Methods

### Setting

Our study was conducted as part of an ongoing quasi-experimental study to evaluate the effectiveness of an educational intervention for HCPs, patients, and hospital officials at three hospitals in Ethiopia: Yekatit 12 Medical College Hospital (Yk 12 MCH), Zewditu Memorial Hospital (ZMH), and Jimma University Medical Center (JUMC). ZMH and YK 12 MC are located in the capital city, Addis Ababa, which has more than 3.3 million inhabitants [24]. JUMC is located 355 km south-west of the capital, in Jimma Zone, which has an estimated total population of 2.4 million [25]. The participating hospitals are comparable regarding years in operation, postoperative settings, and infrastructure (S1 Table).

### Design

We used a qualitative description design [22,23] to explore barriers to and facilitators of QPM in the postsurgical patients. The perspectives of HCPs, patients, and hospital officials were recorded in face-to-face, semi-structured interviews, conducted from October 4, 2016 to December 8, 2016. The method of qualitative description has been used widely in qualitative health research [22], including research on pain management practices [26].

### Sampling and recruitment

The hospitals participating in the main longitudinal, quantitative, quasi-experimental study were also selected for this qualitative study. Purposive sampling with the maximum variation technique was used to select participants. The framework for maximum variation was based on baseline pain intensity (patients with mild, moderate, and severe pain), type of surgery (patients who had undergone orthopedic, gynecologic, and general surgical procedures), and sex. For instance, if one female gynecologic patient with mild pain, one female orthopedic patient with moderate pain, and one male patient with moderate pain who had undergone general surgery were selected from one of the hospitals, two orthopedic male patients with severe pain were selected from the other hospital, and so on. This was continued until the predetermined a priori sample of nine patients was obtained. In the same manner, first we defined a minimum of nine HCPs a priori (including at least two nurses, two surgeons, two gynecologists, and two anesthetists) with as even a representation as possible of men and women and number of service years (see Table 1 in results section). Nurses, surgeons, gynecologists, and anesthetists were included because these HCPs routinely participate in the perioperative pain management of surgical patients in Ethiopia. We also determined that each hospital should contribute at least two HCPs to the sample. To select hospital officials, we considered individuals from two groups, i.e. those who were in charge of the overall administration of the hospitals and those who were in charge of the surgical wards. We then identified potential participants (at least two officials from each hospital). Primarily, researcher (MTE) screened clinical record files to identify eligible patients. Then, patients were approached, and when agreed to be part of the study they completed a brief questionnaire that included demographic information, type of surgery and pain intensity. To recruit HCPs the researcher was present in the surgical wards at office hours and approached surgeons, gynecologists and nurses happen to be caring for the surgical patient. However, anesthetists were approached during their lunch break in the operation theatre’s cafeteria. HCPs who fulfil the selection criteria and agreed to be part of the study completed demographics, background profession, and year of service. To recruit officials the researcher visited them at their office, and scheduled an appointment through the secretary by explaining the purpose of the study. The researcher was then contacted back by the secretary of the officials thorough mobile phone to conduct the interview. The officials also completed an information sheet that included position title, year of service and demographics. Everyone (patients, HCPs and hospital officials) who was invited to participate in the study agreed to do so, and before commencing the semi-structured interview a signed informed conscent was obtained. Table 1 (see results section) presents the demographic and professional characteristics of the participants. After completing the interviews of the initial sample defined above (24 participants), we started to analyze the data to obtain more information on the gathered data to help us select the next participants. However, this analysis showed that the completed interviews had reached data saturation, i.e. the interviews no longer provided new information or voices [27], so that further interviews were deemed unnecessary. The aim of our sampling approach was to help us reach data saturation, which should be considered a priori [27]; a large sample size does not necessarily ensure data saturation [27,28].

**Table 1:** Sociodemographic and clinical characteristics of participants.

**Patient ID**	**Age**	**Sex**	**Type of surgery**	**Pain intensity**^**‡**^	**Hospital**	Duration of interview*
1	32	Female	Orthopedic	1	1	13
2	44	Female	Gynecologic	5	3	14
3	36	Male	General	8	2	17
4	50	Female	General	7	1	17
5	50	Male	General	5	1	14
6	61	Male	Orthopedic	9	2	16
7	23	Male	Orthopedic	9	2	15
8	35	Female	Gynecologic	4	3	13
9	33	Female	Gynecologic	6	3	16
^**†**^**HCP ID**	**Age**	**Sex**	**Profession**	**Years of service**	**Hospital**	Duration of interview*
91	57	Male	Anesthetist	25	2	29
21	37	Female	Nurse	7	3	28
31	44	Male	Nurse	9	1	29
14	43	Male	Nurse	10	2	28
51	35	Female	Surgeon	3	3	25
16	40	Female	Surgeon	7	1	27
17	45	Male	Anesthetist	12	3	22
81	36	Female	Gynecologist	5	1	32
19	44	Male	Gynecologist	8	2	20
**Official ID**	**Age**	**Gender**	**Position**	**Year of service**	**Hospital**	Duration of interview*
21	48	Male	Nursing unit director director	6	1	27
22	33	Male	Medical director	4	3	25
32	35	Male	Clinical director	3	1	26
24	33	Male	General Provost	4	2	32
52	36	Female	Matrons office head	4	2	23
26	34	Male	General Dean	3	2	33

Healthcare professional

‡measured on a numeric rating scale (0–10)

*Numbers are rounded

### Data collection

We collected data during individual, face-to-face semi-structured interviews that lasted on average 15 to 30 minutes. All patient interviews were conducted at the place of their preferences, that is, the bedside, on the hospital wards 24 hours after surgery. Some patients’ were inevitably immobile (e.g, orthopedic patients). Before the interview was started HCPs were informed that the ward door will remain locked for the duration of the interview. Family members were also agreed to leave the room for the duration of the interview. In cases where more than one patient was available in the patient wards, care has been taken to make sure the conversation remained laudable only for the patient and the interviewer. HCPs and hospital officials were interviewed in their respective offices. Interviews were conducted by the first author (MTE), a male lecturer and anesthetist with experience of working with postoperative patients; MTE received the necessary training in qualitative research as part of his PhD curriculum. The interviews were conducted in the local language, Amharic, and were audio recorded. We developed a semi-structured interview guide (S1 File) on the basis of a literature review [29,30] and the study objectives to standardize the interviews [31]. The guide for the patient interviews covered the following areas: patients’ experience of pain after surgery, the perception of pain treatment options, coping mechanisms, perceived barriers to QPM, and an evaluation of the HCPs’ help in alleviating pain. The guide for the interviews with the HCPs and hospital officials included the perceived quality of pain management and barriers to and facilitators of QPM. In addition, the interview guide for hospital officials also included questions about monitoring pain management practices, the availability of the necessary drugs and human resources for pain management, and the policy or standards on how HCPs should manage postoperative pain. The interview guide served only as an outline with the aim to generate a discussion that would help to address the research question, i.e. what are the barriers to and facilitators of quality pain management in surgical patients from the perspectives of patients, healthcare professionals, and hospital officials. The interviewer asked probing questions, such as “What do you mean by that?” and “Can you elaborate more on that, please?” At the end of each interview, the researcher asked the participants to discuss anything else they considered relevant. In line with the proper practice of semi-structured interviewing [31], the interviewer attempted to remain as objective as possible during the interview process. He maintained a good rapport (trust and respect) throughout the interview.

### Ethical considerations

The study was approved by the Jimma University Institutional Review Board, the Medical Ethics Committee of the Ludwig-Maximilians-Universität Munich, and by all participating hospitals. Before conducting the interviews, the interviewer briefly explained the study, including the risks and benefits of participation. The interview continued only after written informed consent was obtained, and participation was voluntary. The interviewer had no prior relationship with any of the participants. The study was performed in accordance with the established ethical standards in the Declaration of Helsinki (1964).

### Data analysis

**Fig 2:**
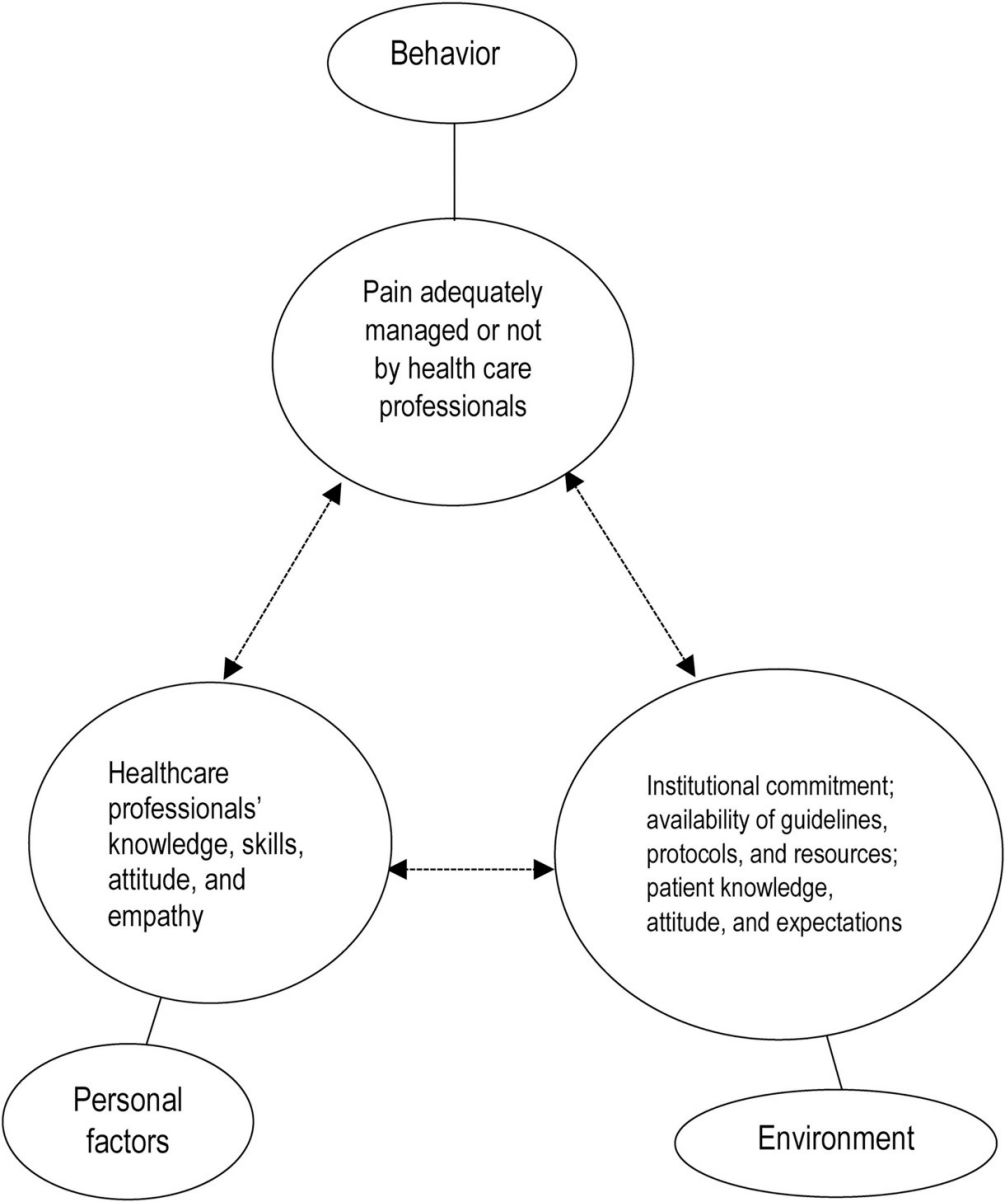
Theoretical framework. Adapted from Wood and Bandura’s Triadic Reciprocal Determinism (Wood and Bandura [54]).

Upon completion of each interview, a complete transcript was produced in Amharic. The interviewer read and reviewed the transcribed data to ensure that they were clear and compared the transcriptions with the original audio recordings for accuracy. Data were analyzed manually by Braun and Clarke’s six-step process of thematic analysis [32]. We used a “bottom-up” approach (inductively) to ensure that important aspects were not missed. Line-by-line coding was performed independently by two authors (MTE, DW), one of whom was a medical sociologist with previous experience in qualitative research (DW). Once duplicate codes had been removed and relevant data extracted, we started searching for themes. In line with the research question, themes were constructed from the codes. Similar themes were collapsed, and some were split, if necessary. The results of the data analysis are presented in Fig 2. Emerging concepts and categories were translated into English by two independent translators. The final English version was established after a discussion between MTE and DW. A third person (an anesthetist) translated the final English version back into Amharic. Finally, a committee of four individuals, consisting of an expert in English (not a co-author of the paper), an anesthetist (not a co-author of the paper), an expert qualitative researcher (DE), and the first author (MTE) settled issues of conceptual and semantic equivalence between the Amharic and the final English versions. The two coders ultimately agreed that, according to the final analysis, the data had reached saturation and no new data or themes were being generated, making additional interviews unnecessary. To increase the dependability of the results, we prepared a detailed study protocol and used two independent coders with different professional backgrounds (MTE and DW) [33]. To further strengthen the confirmability and credibility of the results, we applied the data source triangulation technique in which we used several groups of surgical ward staff working in different hospitals and performing different roles [34] The application of stratified purposive sampling and maximum variation sampling also enhanced the transferability of the results [35].

## Findings

### Study participants

Twenty-four participants were interviewed. The sociodemographic and clinical characteristics and interview durations are summarized in Table 1.

Emerging themes were classified as barriers and facilitators related to HCPs, patients, and the health care system. These themes and respective subthemes are presented below, together with example quotes.

### Barriers related to healthcare professionals

All participants identified HCPs’ lack of empathy and lack of education as barriers to QPM. From the perspective of the HCPs and hospital officials, QPM was hindered by the failure to use pain rating scales to assess patients’ pain intensity and by the fear of side effects and dependence.

#### Lack of empathy

The feeling that “I am on my own” was the thought most commonly shared by patients in the interviews. Patients frequently expressed how they felt neglected by the HCPs, who paid no attention to their level of pain after surgery. In the patients’ view, the HCPs seemed to have little interest in the patients’ wellbeing and not be willing to listen to the patients or treat pain after surgery.

*“Professionals should put themselves in our shoes*. *Whether the wound is big or small*, *it does not matter*, *the pain is the same to us*. *They [professionals] always say it’s ok*, *this is small*. *Doctors should be able to communicate with us…you know…we should be close to them*. *Professionals should have the attitude of servants*, *not masters*. *They [professionals] have to show us compassion*.*” (Hospital 1*, *patient [prostatectomy]*, *male*, *50)*

Also, HCPs and hospital officials admitted a lack of empathy with patients in pain after surgery, and most HCPs had not yet undergone surgical interventions themselves.

*“Because we (professionals) never went through the operation*, *most of us have no idea what it is like to be in pain*. *What can you do*? *You cannot cut and suture them [professionals]*? *It is the way it is*. *Pain is related to experience*; *they don’t have the experience*, *so they will not cope with it*.*” (Hospital 2*, *HCP [anesthetist]*, *male*, *57)*

#### Lack of information about pain and its management during medical education

Most professionals said that the topic of pain was neglected in undergraduate medical education, which strongly emphasized infections and other medical problems.

*“If one patient does not receive proper pain treatment*, *they [professionals] don’t understand the consequences*. *Then the patient suffers*, *develops chronic pain and is discharged with the pain*. *He will eventually return with pain as a complaint*. *Nobody will find the pain because you cannot find it in the laboratory*. *So*, *most likely he will end up in the psychiatric wards*.*” (Hospital 2*, *HCP* ***[****gynecologist]*, *male*, *44****)***

The duration of and access to in-service training were also not perceived as being satisfactory.

“*For example*, *there are 500 nurses*, *and only 50 are selected for the training*. *Then*, *it is declared that the training has been given to all professionals*. *Moreover*, *the trained professional does not share what he or she learned from the training with the rest of the team*. *It is much better if the training includes all the nurses who are part of the care team” (Hospital 2*, *HCP [nurse]*, *male*, *44)*

#### No use of pain rating scales in clinical practice

HCPs and hospital officials frequently mentioned that pain rating scales were not used. They stated that most of them measured pain subjectively, instead of using a standard pain rating scale. HCPs mentioned using patients’ facial expressions and “general condition,” as they put it, to evaluate patients’ level of pain and make a decision about administering analgesics.

*“We take into consideration the type of surgery when giving analgesics*. *Most of the time*, *if the patient underwent thoracic surgery or had a bone fracture*, *we will use strong analgesics*, *if available*. *If it is an abdominal surgery*, *these are less painful*, *so we use weaker analgesics*. *We then follow the patients to see if they are complaining about pain*. *This is critical*.*……*.*this is to identify whether the pain is from the surgery itself or whether it is something else……*.*…like infection development or wound healing………you just have to take patients’ general condition and facial expressions into consideration to decide how severe their pain is*. *This what we use to measure pain in our setting*.*” (Hospital 3*, *HCP [surgeon]*, *male*, *35)*

The way in which the patient asks for pain medication is also crucial in HCPs’ decision whether or not the patient is in pain.

*“The way the patient asks for analgesics matters*. *Some exaggerate the smallest pain*, *while others bear the unbearable…If the patient nags you the whole day and complains a lot*, *we then ask his surgeon and senior physicians to respond*.*” (Hospital 3*, *HCP [nurse]*, *female*, *37)*

#### Fear of side effects and dependence

HCPs were afraid of opioid-related side effects, in particular with respect to legal issues. To be on the “safe side” and avoid accountability, they mainly relied on nonsteroidal anti-inflammatory drugs (NSAIDs), despite the knowledge about their limited efficacy.

*“Narcotics are not available like other analgesics*, *but even if they are available*, *there is a worry*. *This worry concerns respiratory depression*, *because of the drugs*. *Professionals have to be on the safe side and avoid legal consequences*, *so they intentionally avoid them*. *Also*, *because these drugs are prone to abuse (addiction) the chance of these drugs reaching the hand of the professional is also rare*.*” (Hospital 3*, *HCP [anesthetist]*, *male*, *45)*

Also, independent of the HCPs attitude towards opioids, most of them believed that it was not wise to give analgesics to the patient every time he or she complained because of the risk of side effects.

*“The surgery is part of the care*, *so there will always be pain*. *Even when the wound starts to heal*, *and the skin begins to close naturally*, *there is pain*. *So*, *every time the patient complains about pain I don’t think it is appropriate to give analgesics*. *Otherwise*, *there will be adverse effects*.*” (Hospital 1*, *hospital official [nursing unit director]*, *48*, *male)*

### Facilitators related to healthcare professionals

Continuing education for HCPs was identified as a facilitator of QPM after surgery. HCPs stressed that the education should be carefully designed to improve their communication skills, cultural competency, and ethical norms to help them provide respectful, compassionate care.

#### Provide in-house/on-the-job training for healthcare professionals

HCPs and hospital officials argued that the lack of emphasis on pain (or ignorance about it) and its management in undergraduate medical and nursing education can be addressed by the hospitals themselves when training young HCPs.

*“If possible*, *we need to intervene in the pre-service education*. *In the same way that we teach them to give anti-malarial drugs for malaria patients*, *they should be able to manage a patient’s pain after an operation*. *Especially during their internship period*, *a lot can be done*. *We need to start regarding pain as a disease*.*” (Hospital 1*, *hospital official [clinical director]*, *male 35)*

#### Enhance the ability of healthcare professionals to create positive rapport with patients

Patients and some HCPs felt that the lack of a harmonious relationship between HCPs and patients affected patients’ mood and emotions. HCPs and hospital officials believed that, in order to create a favorable caring environment for the patient, HCPs should receive training in ethics and psychology, in addition to training in the physiology and pharmacology of pain.

“*Patients are not mere bone and flesh*. *They have moods and emotions*. *I think pain management should start with this attitude*. *They are in pain*. *You don’t have to be an additional cause*. *You need to be considerate*, *and the best way to achieve this is to teach medical professionals about ethics*, *norms and compassionate care in addition to the usual anatomy and physiology*.” (*Hospital 2*, *HCP [anesthetist]*, *male*, *57*)

#### Increase the cultural competence of professionals

HCPs and hospital officials recommended adding cultural competency to the pain education curriculum because Ethiopian society consists of many different ethnic groups.

*“We need to increase the cultural competency of professionals*. *They have to know in detail for whom they are caring and who they are trying to cure*. *They should be familiar with their way of life*, *how they perceive*, *react to and treat pain*. *We are so diverse in culture and language*, *with…uh…about 83 different languages and 200 different dialects*.*” (Hospital 3*, *hospital official [medical director]*, *male*, *33)*

### Barriers related to patients

The socially anchored attitude towards pain and patients’ attitudes towards analgesics and dealing with pain rather than asking for relief were identified as barriers to QPM.

#### Positive social appraisal of ability to cope with pain

Professionals stated that being able to cope with pain is usually viewed positively by people. Before patients are admitted to hospital, disease management commonly includes painful techniques, such as applying a red-hot iron to the skin. According to hospital officials, by focusing on the disease such traditions have played a large role in undermining pain treatment and making patients disregard pain.

*“Our society usually*, *when suffering from different diseases [pain]*, *uses a red-hot iron that is applied to the skin*. *Besides*, *they don’t ask for analgesics*, *even if they want to*, *because they feel that the doctors or nurses might not view this behavior positively and it might end up affecting their relationship with the professionals and ultimately their care*.*” (Hospital 3*, *hospital official [medical director]*, *male*, *33)*

Most patients perceived postoperative pain as something simple and temporary that would go away with time and healing. They stated that, instead of worrying about the pain, they were preoccupied with wound healing and returning home as quickly as possible.

*“I have no idea*. *I let them [professionals] do as they wish to do*. *Also*, *they told me it’s minor pain*. *So*, *I didn’t care too much*. *I just want to heal and go back home*.*” (Hospital 2*, *patient [cholecystectomy]*, *male*, *36)*

#### Dealing with pain

Patients also preferred to tolerate and deal with even severe pain rather than use analgesics.

*“When I have pain*, *I forcefully close my eyes and sleep…I don’t ask for analgesics* …*oh…uh… because I don’t know… they [professionals] also told me it is a minor procedure*, *so I did not pay attention to it*.*” (Hospital 2*, *patient [open reduction internal fixation]*, *male*, *61)*

This idea was also found in some of the responses given by the participating HCPs. They stated that pain was not an alarming sign compared to other signs of infection, for example.

*“Our patients*, *actually we [professionals] and our people in general*, *can [try to] bear pain*. *For example*, *when someone says I have a fever and I have pain*, *we don’t react the same way*. *When you hear someone has a fever you tremble*, *if it is a pain you just take it lightly*.*” (Hospital 2*, *hospital official [deputy matrons office head]*, *male*, *36)*

According to the HCPs, this kind of tradition was established in the hospital setting a long time ago.

*“There has been an existing trend for a long time that a patient has to be able to beat the pain and professionals will not respond quickly if the patient is in pain and groaning*.*” (Hospital 1*, *HCP [surgeon]*, *female*, *40)*

HCPs stated that patients also liked to wait for the pain to go away by itself or for the wound to heal completely rather than depend on analgesics.

*“Sometimes*, *they withstand the pain and say it will go away by itself*. *They don’t want to take drugs*, *especially those with a previous surgery history*. *They prefer to cope with it in their own way*.*” (Hospital 1*, *HCP [gynecologist]*, *female*, *36)*

#### Analgesics do not heal the wound

Some patients did not believe that analgesics were any help in healing the injury. They did not take analgesics because they would only take away the pain but would not cure the disease (wound).

*“They give me analgesics; I feel ok*, *then after a while I will again feel pain*. *Pain will not go away with drugs*. *You feel better when the wound heals*.*” (Hospital 3*, *patient [myomectomy]*, *female*, *35)*

#### Fear of side effects and dependence

Similar to HCPs, patients were afraid of the side effects of analgesics. Many would prefer not to take any drugs because of concerns about developing dependence or other side effects. In most patients’ view, analgesics have many complications and side effects and therefore it is better to recover without the help of drugs.

*“I don’t want my body to depend at all on drugs to heal*. *It is better to move around and forget about the pain than to take drugs every time you feel pain*.*” (Hospital 1*, *patient [mastectomy]*, *female*, *50)*

The HCPs also stated that the patients’ fear of side effects was sometimes a significant challenge when they wanted to treat the patients’ pain.

*“There are occasions when we offer pain medication and the patient themselves refuses because of a fear of addiction and side effects……even while the patient is in severe pain*, *they refuse to take drugs because of a fear of side effects*.*” (Hospital 2*, *HCP [nurse]*, *male*, *44)*

### Facilitators related to patients

Both HCPs and hospital officials agreed that educating patients could facilitate the provision of QPM.

#### Patient education

The typical response given by HCPs was that patient education would be important to improve QPM in the postoperative period. Most importantly, patients should consider pain management their right and should not hesitate to demand and insist on pain treatment.

*“Patients should say*, *“anti-pain treatment is my right*!*” They need to be trained*, *should be familiar with pain rating scales and encouraged to tell their feelings without any hesitation*. *Because most of them [patients] believe this to be part of the care*, *they tend to beat/bear the pain*; *we should first and foremost discourage such behavior*.*” (Hospital 1*, *HCP [surgeon]*, *female*, *40)*

### Barriers related to the health care system

Low physician- and nurse-to-patient ratios, a lack of resources, and health policy itself were identified as health care system-related obstacles to QPM.

#### Ratio of healthcare professionals to patients

The physician- and nurse-to-patient ratios were among the most frequently mentioned barriers. Both hospital officials and HCPs stated that only a small number of HCPs were available on the wards for a large number of patients.

*“On the ward*, *there might be 40*, *50 patients*, *and there are only 5 or 4 nurses*. *Imagine*, *how could you give better care …because of work overload you feel weary*. *When you work for many years*, *this leads to exhaustion and wears you down*.*” (Hospital 1*, *HCP [nurse]*, *female*, *37)*

#### Availability of resources

The high costs of narcotics and the lack of opioids were further significant challenges mentioned by the HCPs and hospital officials.

*“Take pethidine*. *It’s around 0*.*50 USD in the hospital pharmacy*, *but at the pharmacy outside it costs about 2*.*8 USD or 3*.*2 USD*. *Especially morphine*, *it’s unthinkable*, *it’s the cheapest analgesic in most other countries*, *from my experience*, *but in Ethiopia*, *a single injection ampule costs about 3*.*8–4*.*0 USD*.*” (Hospital 2*, *HCP [anesthetist]*, *male*, *57)*

#### Health care priorities

Hospital officials mentioned that Ethiopia’s health policy should pay as much attention to postoperative pain as it does to microbial infections and other infectious diseases.

*“There is a pain-free initiative just initiated by the ministry of health*. *This should be strengthened and continued*. *The commitment shown to infectious diseases should be extended to pain also*.*” (Hospital 2*, *hospital official [general dean]*, *male*, *34)*

### Facilitators related to the health care system

Strict supervision of post-surgical pain management, an adequate supply of drugs, and the establishment of treatment protocols and standards of care were major recommendations by the HCPs. Hospital officials, on the other hand, recognized that an adequate supply of analgesics, continuous supervision, and the establishment of policies and standards of care were crucial factors.

#### Strict supervision of trainees in providing postoperative pain management

There was a demand for clear and even legal consequences if a patient’s pain is undertreated or untreated. Participants also stressed that HCPs who do not provide responsible care should be held accountable and responsible and that a task force should be established to develop a treatment guideline.

*“We need to have a clear policy of pain management in the hospital*. *That way you can influence professionals to be serious about it*. *And then you can hold responsible anyone who does not abide [by the policy]… there should also be a multi-disciplinary task force that researches the issue in detail and develops a guideline*.*” (Hospital 2*, *hospital official [general dean]*, *male*, *34)*

#### Provision of adequate drugs

Improvement of the supply of analgesics (type and quantity) was the facilitator of QPM most frequently suggested by HCPs and hospital officials. The latter also suggested financial and budgetary support, which should explicitly aim to establish standard QPM.

*“There are drugs that are not even available on the market*. *They should be available*. *The country should also make sure these drugs are in the essential drug list…*..*some drugs are not being brought in by the ministry of health*. *We don’t have easy access to these drugs; we should*.*” (Hospital 1*, *HCP [surgeon]*, *female*, *40)*

#### Establishment of a guideline for quality pain management

HCPs and hospital officials suggested that the health care system should be involved in postoperative pain management because it directly affects the outcome of surgical patients. There should be a clear guideline that states explicitly how postoperative pain should be managed in hospitals.

*“Advocacy is the most important thing*, *but as a health system we should be able to develop a protocol and establish a policy…*.*” (Hospital 1*, *hospital official [clinical director]*, *male*, *35)*

## Discussion

The aim of this study was to explore the perspectives of post-surgical patients, HCPs, and hospital officials on the barriers to and facilitators of the effective delivery of QPM services. To our knowledge, this is the first multicenter study to qualitatively evaluate issues related to postoperative QPM in Ethiopia. This report provides unique information by incorporating the views of patients, HCPs, and hospital officials. Findings from such a variety of perspectives can inform the design and implementation of strategies to improve the delivery of QPM services to surgical patients. This is especially important in LMIC, where access to adequate treatment has been reported to be limited [36].

In general, the patients in our study felt that HCPs’ lack of empathy was the main reason for undertreatment of postoperative pain. The HCPs agreed with this view and associated the lack of empathy with burnout resulting from the low ratio of professionals to patients on the wards. Indeed, a recent systematic review of cross-sectional studies confirmed a negative correlation between burnout and empathy [37], although the authors still argued that it is rather difficult to establish causality from such an observational study. A previous report from the same setting (Ethiopia) described low emotional and cognitive empathy scores among medical students, which seems to support the perspective of the patients in our study [38]. Whatever causes the lack of empathy, it undoubtedly prevents the HCPs from understanding the patients’ pain and therefore should be addressed by the health care system.

Patients also seemed in part not to be aware of the danger of untreated/undertreated postoperative pain and to perceive postoperative pain as a natural consequence of surgery. They regarded it as a minor phenomenon that would go away with time and tissue healing, without any long-term consequences. Patients’ belief that pain is “not harmful” has previously been identified as a significant barrier to QPM [39]. Because HCPs not only supported but also endorsed this belief, it became common among patients. Surprisingly, other studies have found that HCPs actually do believe that postoperative pain is a short-term phenomenon and decreases with time and tissue healing [39]. This is inconsistent with the substantial evidence that postoperative pain has long-lasting adverse effects, which are caused by sensitization of the peripheral and central nervous systems [40]. Also, patients are not surprised when they experience pain after surgery and instead attempt to cope with it on their own without the help of analgesics. Patients see analgesics as the last resort, should the pain become unbearable. They believe that it is better to avoid painkillers as much as possible and remain in pain. This “pain by choice” phenomenon also seems to be a socially desirable behavior. The avoidance of analgesics in post-surgical patients was reported as early as two decades ago [41] and is a barrier to pain treatment worldwide [42]. For these reasons, preoperative patient education has been recommended for several years as part of routine care to improve postoperative pain management [39], and these efforts have been successful [41]. However, preoperative patient education was not part of the routine care in any of the hospitals participating in this study.

HCPs also stated that they do not use standardized pain scales to determine whether a patient is in pain or not. Instead, they assess the level of pain on the basis of the patient’s facial expression and the nature and type of surgery. Early studies highlighted that relying on patient self-reports or HCPs’ personal evaluations of facial expressions and crying or moaning was a significant barrier to postoperative pain management in both developed countries [43] and LMIC [44].

All these mistaken beliefs about postoperative pain are the result of HCPs’ poor knowledge and skills and their attitude. A lack of education and training is the most common barrier to pain management identified by previous studies, particularly in low-resource settings, where there is a greater lack of individuals properly trained in pain management than in high-resource settings [10]. Hospital officials also felt that this education gap is due to a lack of emphasis on pain education in the Ethiopian medical and nursing curricula. Furthermore, they stressed that most undergraduate and even postgraduate medical and nursing curricula focus on infectious and other “important” diseases and pay little attention to pain. These statements make clear that Ethiopian hospitals do not prioritize pain management; this is also the case in Iran [45]. The absence of pain education in medical, pharmacy, and nursing curricula has been highlighted previously as an obstacle to pain management in surgical patients [46]. This is especially true in Ethiopia, where a nationwide study confirmed that HCPs were not prepared to assess and treat pain in general [14]. In addition, resource-based limitations, such as the lack of strong analgesics like opioids, prevents HCPs from delivering QPM. A very recent report from Rwanda also stated that the lack of resources was the most frequently identified barrier to adequate treatment of postoperative pain [47]. In Africa, a lack of resources has prevented the health care system from delivering QPM for several years [48].

Regarding factors that facilitate provision of QPM, participants suggested providing in-house/on-the-job training for HCPs as a first step. Hospital officials also felt that HCPs’ education should include topics that could enhance their cultural competency and skills and thus allow them to create a good rapport with patients. Participating HCPs said that patient education should also be part of the intervention. A randomized controlled study recommended preoperative patient information as a tool to decrease the intensity of patients’ postoperative pain and increase patient satisfaction [49].

The most common facilitator suggested by participating HCPs and hospital officials was the establishment of guidelines, protocols, and accountability. Global evidence supports the development and implementation of guidelines for high-quality health care [50]. Establishing policies, guidelines, and protocols has been recommended for improving postoperative pain management in low-resource settings in particular [51]. Previous studies have confirmed that guidelines can help to hold HCPs accountable for inadequate care [52], and the absence of guidelines allows HCPs to ignore postoperative pain management because the undertreatment of pain has no consequences. Hence, hospital officials believed that strict supervision of trainees in providing postoperative pain management and the provision of adequate drugs are also critical. All participants believed that protocols and guidelines should be established for treating postoperative pain. Professionals also stated that people in managerial and leadership positions should make analgesics available and design policies to reward the desired behavior, for example through praise and recognition.

Our findings suggest that to achieve sustainable improvement of postoperative pain management the whole of Ethiopian society needs to change their attitude to postoperative pain. Social norms can be systematically changed by educating patients and their families, for example. When HCPs face a demanding and aware patient, they will be forced to change their behavior [20].

The above-mentioned barriers and facilitators can be best understood on the basis of Albert Bandura’s theoretical framework of reciprocal determinism. According to reciprocal determinism, any human behavior is the result of external environmental factors (via social stimulus events) and internal personal factors (through cognitive processes) [21,53]. In our study, the internal personal factors included HCPs’ lack of empathy, lack of education on pain assessment and treatment, and fear of side effects and dependence. The environmental factors included the social milieu with which HCPs continually interact (e.g., patients’ attitudes towards pain and analgesics) and the surgical ward environment (e.g., availability of resources, protocols, guidelines, regulation, ratio of professionals to patient). HCPs’ poor practice with regard to postoperative pain management was reciprocally (bi-directionally) affected by these personal and environmental factors, as shown in Fig 1. In our study, the social environment, which included factors such as patients’ willingness to suffer pain by avoiding analgesics and their inclination to deal with pain and underestimate its consequences, was likely to encourage HCPs to disregard patients’ pain. Similarly, HCPs did not have a sense of accountability because protocols and guidelines were lacking. Hence, the environment was friendly to those HCPs who lacked empathy, ignored postoperative pain management and had a negative attitude towards pain management, which in turn caused patients to suffer. As regards the personal factors, HCPs may have lacked knowledge and skills in pain management and have had the wrong attitude towards it because they did not receive appropriate training; this in turn may have led to them to have false beliefs and poor practices, which again would result in patients suffering.

Future interventions should carefully consider these SCT perspectives. For example, if an intervention only targets HCPs, it might be unsuccessful because of external barriers, such as those relating to patients and the environment. Because barriers to and facilitators of postoperative pain management interact with one another, a multi-faceted intervention aimed at HCPs, patients, and the health system as a whole is more likely to be successful.

## Limitations

Our results might not be generalizable to all surgical patients in all parts of Ethiopia because we included only patients undergoing elective general, gynecologic, or orthopedic surgery. Furthermore, this was not a quantitative study, i.e. there were no specific power calculations or assessments of statistical significance or effect size. Cultural, religious, and contextual differences in multi-ethnic countries such as Ethiopia could influence the results. Moreover, the validity might have been compromised because the interview transcripts were not shown to the participants. Although we used the reciprocal determinism theory to explain the reciprocal influence of the environment and personal factors on the HCPs’ practice of pain management, we did not specifically examine the individual constructs of SCT and did not measure the HCPs’ performance. The fact that the patient interviews were short (mean duration: 15 minutes) potentially limits the richness of the data and depth of the analysis. All patients preferred the bedside for the interview and the presence of other patients in the wards might have affected their response. However, patients were very comfortable throughout the interview and which might neutralize the possible bias. By using purposive and maximum variation sampling to include a diverse group of participants, however, we may have counteracted this limitation, as indicated by the fact that our findings are in line with previous reports [45,47]. Nevertheless, we cannot guarantee that by using these sampling techniques we included a wide enough variety of people to obtain information on all relevant barriers to and facilitators of QPM, i.e. to reach data saturation. Despite these limitations, to our knowledge the qualitative work presented here was the first multi-center study to obtain perspectives on pain management in Ethiopia from various groups. To date, only a few qualitative studies have used reciprocal determinism to explain barriers to and facilitators of QPM in the surgical patients.

## Conclusions

This study illustrates the barriers to and facilitators of postoperative pain management from the perspective of Ethiopian patients, HCPs, and hospital officials. The findings provide probably the first qualitative insight into the factors that affect the management of postoperative pain in Ethiopia. Participants in this study believed that QPM is difficult under the current conditions, as a result of the above-mentioned barriers. From the perspective of the reciprocal determinism theory, the HCPs’ current poor provision of QPM is in part related to the HCPs themselves, i.e. to personal factors that are a result of poor training during their medical education and while working. In addition, patients’ attitudes and the lack of political will to make changes to the health care system (i.e. failure to make resources available and establish guidelines and lack of leaders’ insight that pain management is a priority compared with other “diseases”) have contributed to creating an environment in which HCPs continue to practice the way they have been practicing in the past. These findings call for holistic and inclusive interventions that target HCPs, patients, and hospital officials. The current situation is unlikely to improve if interventions educate only HCPs about pain physiology, pharmacology, and management and do not include the other stakeholders. To achieve high-quality postoperative pain management, we also have to educate patients and modify the environment.

## Supporting information

S1 Table Characteristics of participating hospitals. (PDF)

S2 Table Consolidated criteria for reporting qualitative research (COREQ): 32-item checklist. (PDF)

S1 File Interview guide. (PDF)

## References

[1] Weiser TG, Haynes AB, Molina G, Lipsitz SR, Esquivel MM, et al (2016) Size and distribution of the global volume of surgery in 2012. World Health Organization Bulletin of the World Health Organization 94: 201 10.2471/BLT.15.159293 26966331 PMC4773932

[2] Lundborg C (2015) Why postoperative pain remains a problem. Journal of pain & palliative care pharmacotherapy 29: 300–302. 26305515 10.3109/15360288.2015.1065940

[3] Gerbershagen HJ, Aduckathil S, van Wijck AJ, Peelen LM, Kalkman CJ, et al (2013) Pain intensity on the first day after surgery: a prospective cohort study comparing 179 surgical procedures. Anesthesiology 118: 934–944. 10.1097/ALN.0b013e31828866b3 23392233

[4] Hetmann F, Kongsgaard UE, Sandvik L, Schou-Bredal I (2015) Prevalence and predictors of persistent post-surgical pain 12 months after thoracotomy. Acta Anaesthesiol Scand 59: 740–748. 10.1111/aas.12532 25907109

[5] Crombie IK, Davies HT, Macrae WA (1998) Cut and thrust: antecedent surgery and trauma among patients attending a chronic pain clinic. Pain 76: 167–171. 9696470

[6] Stephens J, Laskin B, Pashos C, Pena B, Wong J (2003) The burden of acute postoperative pain and the potential role of the COX-2-specific inhibitors. Rheumatology (Oxford) 42 Suppl 3: iii40–52. 14585917 10.1093/rheumatology/keg497

[7] King NB, Fraser V (2013) Untreated Pain, Narcotics Regulation, and Global Health Ideologies. PLOS Medicine 10: e1001411 10.1371/journal.pmed.1001411 23565063 PMC3614505

[8] Joshi GP, Ogunnaike BO (2005) Consequences of inadequate postoperative pain relief and chronic persistent postoperative pain. Anesthesiol Clin North America 23: 21–36. 10.1016/j.atc.2004.11.013 15763409

[9] Murray AA, Retief FW (2016) Acute postoperative pain in 1 231 patients at a developing country referral hospital: incidence and risk factors. Southern African Journal of Anaesthesia and Analgesia 22: 19–24.

[10] (2011) Managing Acute Pain in the Developing World International Association for the Study of Pain.

[11] Zoëga S, Sveinsdottir H, Sigurdsson GH, Aspelund T, Ward SE, et al (2015) Quality pain management in the hospital setting from the patient's perspective. Pain Practice 15: 236–246. 10.1111/papr.12166 24433333

[12] Gordon DB, Pellino TA, Miaskowski C, McNeill JA, Paice JA, et al (2002) A 10-year review of quality improvement monitoring in pain management: recommendations for standardized outcome measures. Pain Manag Nurs 3: 116–130. 10.1053/jpmn.2002.127570 12454804

[13] Chou R, Gordon DB, de Leon-Casasola OA, Rosenberg JM, Bickler S, et al (2016) Management of Postoperative Pain: a clinical practice guideline from the American pain society, the American Society of Regional Anesthesia and Pain Medicine, and the American Society of Anesthesiologists' committee on regional anesthesia, executive committee, and administrative council. The Journal of Pain 17: 131–157. 10.1016/j.jpain.2015.12.008 26827847

[14] Centers for Disease Control and Prevention FDRoEMoH, Ethiopian Public Health Association (April 2011) Baseline Evaluation of Pain Management Practices and Teaching in Health Facilities and Health Training Schools in Ethiopia 66–67 p.

[15] Woldehaimanot TE, Eshetie TC, Kerie MW (2014) Postoperative pain management among surgically treated patients in an Ethiopian hospital. PloS one 9: e102835 10.1371/journal.pone.0102835 25033399 PMC4102595

[16] Cleary J, Powell RA, Munene G, Mwangi-Powell FN, Luyirika E, et al (2013) Formulary availability and regulatory barriers to accessibility of opioids for cancer pain in Africa: a report from the Global Opioid Policy Initiative (GOPI). Ann Oncol 24 Suppl 11: xi14–23. 24285225 10.1093/annonc/mdt499

[17] Ethiopian Publich Health Association FDRoEMoH, Center for Disease Control and Prevention (April 2011) Baseline Evaluation of Pain Management Practices and Teaching in Health Facilities and Health Training Schools in Ethiopia.

[18] Chao TE, Burdic M, Ganjawalla K, Derbew M, Keshian C, et al (2012) Survey of surgery and anesthesia infrastructure in Ethiopia. World J Surg 36: 2545–2553. 10.1007/s00268-012-1729-3 22851147

[19] Alzghoul BI, Abdullah NAC (2015) Psychosocial theories and pain management practices: A review of empirical research. Mediterranean Journal of Social Sciences 6: 60.

[20] Bandura A (1999) Social cognitive theory of personality. Handbook of personality: Theory and research: 154–196.

[21] Bandura A (1999) Social cognitive theory: An agentic perspective. Asian journal of social psychology 2: 21–41. 10.1146/annurev.psych.52.1.1 11148297

[22] Sandelowski M (2010) What's in a name? Qualitative description revisited. Res Nurs Health 33: 77–84. 10.1002/nur.20362 20014004

[23] Neergaard MA, Olesen F, Andersen RS, Sondergaard J (2009) Qualitative description—the poor cousin of health research? BMC Med Res Methodol 9: 52 10.1186/1471-2288-9-52 19607668 PMC2717117

[24] Agency CI The WORLD FACT BOOK.

[25] Ethiopia CSAo (2007) The 2007 Population and Housing Census of Ethiopia: Statistical Report for Oromiya Region; Part I: Population Size and Characteristics. 2–3 p.

[26] Hadi MA, Alldred DP, Briggs M, Marczewski K, Closs SJ (2017) 'Treated as a number, not treated as a person': a qualitative exploration of the perceived barriers to effective pain management of patients with chronic pain. BMJ Open 7. 10.1136/bmjopen-2017-016454 PMC5541634 28606909

[27] Fusch PI, Ness LR (2015) Are we there yet? Data saturation in qualitative research. The Qualitative Report 20: 1408.

[28] Burmeister E, Aitken LM (2012) Sample size: how many is enough? Aust Crit Care 25: 271–274. 10.1016/j.aucc.2012.07.002 22835279

[29] Rejeh N, Ahmadi F, Mohammadi E, Kazemnejad A, Anoosheh M (2009) Nurses' experiences and perceptions of influencing barriers to postoperative pain management. Scand J Caring Sci 23: 274–281. 10.1111/j.1471-6712.2008.00619.x 19645804

[30] N. R, F. A, E. M, M. A, A. K (2008) Barriers to, and facilitators of post‐operative pain management in Iranian nursing: a qualitative research study. International Nursing Review 55: 468–475. 10.1111/j.1466-7657.2008.00659.x 19146560

[31] DiCicco-Bloom B, Crabtree BF (2006) The qualitative research interview. Medical Education 40: 314–321. 10.1111/j.1365-2929.2006.02418.x 16573666

[32] Braun V, Clarke V (2006) Using thematic analysis in psychology. Qualitative Research in Psychology 3: 77–101.

[33] Lincoln YS, Guba EG (1986) But is it rigorous? Trustworthiness and authenticity in naturalistic evaluation. New directions for program evaluation 1986: 73–84.

[34] Shenton AK (2004) Strategies for ensuring trustworthiness in qualitative research projects. Education for information 22: 63–75.

[35] Forero R, Nahidi S, De Costa J, Mohsin M, Fitzgerald G, et al (2018) Application of four-dimension criteria to assess rigour of qualitative research in emergency medicine. BMC health services research 18: 120 10.1186/s12913-018-2915-2 29454350 PMC5816375

[36] Goucke CR, Chaudakshetrin P (2018) Pain: A Neglected Problem in the Low-Resource Setting. Anesth Analg 126: 1283–1286. 10.1213/ANE.0000000000002736 29547421

[37] Wilkinson H, Whittington R, Perry L, Eames C (2017) Examining the relationship between burnout and empathy in healthcare professionals: A systematic review. Burnout Research 6: 18–29. 10.1016/j.burn.2017.06.003 28868237 PMC5534210

[38] Dehning S, Girma E, Gasperi S, Meyer S, Tesfaye M, et al (2012) Comparative cross-sectional study of empathy among first year and final year medical students in Jimma University, Ethiopia: Steady state of the heart and opening of the eyes. BMC Medical Education 12: 34 10.1186/1472-6920-12-34 22624580 PMC3432612

[39] Carr E (2007) Barriers to effective pain management. The Journal of Perioperative Practice 17: 200 10.1177/175045890701700502 17542389

[40] Wilder-Smith OH, Tassonyi E, Arendt-Nielsen L (2002) Preoperative back pain is associated with diverse manifestations of central neuroplasticity. Pain 97: 189–194. 12044615 10.1016/S0304-3959(01)00430-4

[41] Wilder-Smith CH, Schuler L (1992) Postoperative analgesia: pain by choice? The influence of patient attitudes and patient education. Pain 50: 257–262. 1454382 10.1016/0304-3959(92)90029-B

[42] Brydon C, Asbury A (1996) Attitudes to pain and pain relief in adult surgical patients. Anaesthesia 51: 279–281. 8712330 10.1111/j.1365-2044.1996.tb13647.x

[43] Nash R, Yates P, Edwards H, Fentiman B, Dewar A, et al (1999) Pain and the administration of analgesia: what nurses say. Journal of Clinical Nursing 8: 180–189. 10401351 10.1046/j.1365-2702.1999.00228.x

[44] Hodges SC, Mijumbi C, Okello M, McCormick BA, Walker IA, et al (2007) Anaesthesia services in developing countries: defining the problems. Anaesthesia 62: 4–11. 10.1111/j.1365-2044.2006.04907.x 17156220

[45] Rejeh N, Ahmadi F, Mohammadi E, Anoosheh M, Kazemnejad A (2008) Barriers to, and facilitators of post-operative pain management in Iranian nursing: a qualitative research study. Int Nurs Rev 55: 468–475. 10.1111/j.1466-7657.2008.00659.x 19146560

[46] Medrzycka-Dabrowska W, Dabrowski S, Basinski A (2015) Problems and Barriers in Ensuring Effective Acute and Post-Operative Pain Management—an International Perspective. Adv Clin Exp Med 24: 905–910. 10.17219/acem/26394 26768644

[47] Nyirigira G, Wilson RA, VanDenKerkhof EG, Goldstein DH, Twagirumugabe T, et al (2018) Barriers and facilitators to postoperative pain management in Rwanda from the perspective of healthcare providers: a contextualization of the Theory of Planned Behaviour. Canadian Journal of Pain. 10.1080/24740527.2018.1451251 PMC8730569 35005369

[48] Johnson AP, Mahaffey R, Egan R, Twagirumugabe T, Parlow JL (2015) Perspectives, perceptions and experiences in postoperative pain management in developing countries: A focus group study conducted in Rwanda. Pain Research & Management: The Journal of the Canadian Pain Society 20: 255–260. 10.1155/2015/297384 PMC4596633 26448971

[49] Al-Azawy M, Oterhals K, Fridlund B, Aßmus J, Schuster P (2015) Premedication and preoperative information reduces pain intensity and increases satisfaction in patients undergoing ablation for atrial fibrillation. A randomised controlled study. Applied Nursing Research 28: 268–273. 10.1016/j.apnr.2015.01.011 26608424

[50] Grimshaw J, Eccles M, Russell I (1995) Developing clinically valid practice guidelines. J Eval Clin Pract 1: 37–48. 9238556 10.1111/j.1365-2753.1995.tb00006.x

[51] Pain IAftSo (2010) Guide to Pain Management in Low-Resource Settings. SEATTLE. 317–320 p.

[52] Tingle J (1997) Clinical guidelines: legal and clinical risk management issues. Br J Nurs 6: 639–641. 10.12968/bjon.1997.6.11.639 9250071

[53] Bandura A (1989) Six theories of child development. Annals of child development 6: 1–60.

[54] Wood R, Bandura A (1989) Social cognitive theory of organizational management. Academy of management Review 14: 361–384.

