# Supplementary material for: The views of patients, healthcare professionals and hospital officials on barriers to and facilitators of quality pain management in Ethiopian hospitals: A qualitative study
Source: PLoS One. 2019 Mar 14;14(3):e0213644. doi: 10.1371/journal.pone.0213644 (PMC6417681; doi:10.1371/journal.pone.0213644)
Supplement: S1 Table — (PDF) [file pone.0213644.s001.pdf]

S1 Table. Characteristics of participating hospitals

Name of the hospital	Year established	Location	Catchment population	Number of beds	Number of professionals in the selected hospitals	Postoperative pain protocol/Guideline
Zewditu Memorial Hospital*	1940 GC	Addis Ababa, Ethiopia	600,000	340	7 gynecologists, 7 surgeons, 40 nurses	No
Yekatit 12 Hospital	1923 GC	Addis Ababa, Ethiopia	4 million	340	6 gynecologists, 10 surgeons, 3 orthopedists, and 50 nurses	No
Jimma University Medical Center	1938 GC	Jimma, Ethiopia	15 million	643	9 surgeons, 8 gynecologists, 2 orthopedists, and 76 nurses	N0

Note: GC = Gregorian calendar, *No orthopedic surgery or orthopedic surgeon is available in the hospital,
