# Supplementary material for: The views of patients, healthcare professionals and hospital officials on barriers to and facilitators of quality pain management in Ethiopian hospitals: A qualitative study
Source: PLoS One. 2019 Mar 14;14(3):e0213644. doi: 10.1371/journal.pone.0213644 (PMC6417681; doi:10.1371/journal.pone.0213644)
Supplement: S2 Table — (PDF) [file pone.0213644.s002.pdf]

S2 Table. Consolidated criteria for reporting qualitative studies (COREQ): 32-item checklist.

Developed from:
Tong A, Sainsbury P, Craig J. Consolidated criteria for reporting qualitative research (COREQ): a 32-item checklist for interviews and focus groups. *International Journal for Quality in Health Care*. 2007. Volume 19, Number 6: pp. 349 – 357

**No. Item**	**Guide questions/description**	**Reported on Page #**
**Domain 1: Research team and reflexivity**		
Personal Characteristics		
1. Inter viewer/facilitator	Which author/s conducted the interview or focus group?	Million Tesfaye Eshete (MTE; page 10)
2. Credentials	What were the researcher's credentials? E.g. PhD, MD	Million Tesfaye Eshete, BSc, MSc, PhD candidate
3. Occupation	What was their occupation at the time of the study?	Lecturer and anesthetist (page 10).
4. Gender	Was the researcher male or female?	Male (page 10)

5. Experience and training	What experience or training did the researcher have?	See Methods (pages 7 through 10) - The researcher has experience of working with postoperative patients and took the necessary training in qualitative research as part of his PhD curriculum.
Relationship with participants		
6. Relationship established	Was a relationship established prior to study commencement?	No (page 11)
7. Participant knowledge of the interviewer	What did the participants know about the researcher? e.g. personal goals, reasons for doing the research	Page 7
8. Interviewer characteristics	What characteristics were reported about the inter viewer/facilitator? e.g. Bias, assumptions, reasons and interests in the research topic	Page 7
**Domain 2: study design**		
Theoretical framework		
9. Methodological orientation and Theory	What methodological orientation was stated to underpin the study? e.g. grounded theory, discourse analysis, ethnography, phenomenology, content analysis	See Methods (pages 7 through 10)
Participant selection		
10. Sampling	How were participants selected? e.g. purposive, convenience, consecutive, snowball	Page 7
11. Method of approach	How were participants approached? e.g. face-to-face, telephone, mail, email	Page 8
12. Sample size	How many participants were in the study?	Page 7
13. Non-participation	How many people refused to participate or dropped out? Reasons?	No one refused (page 9).
Setting		
14. Setting of data collection	Where was the data collected? e.g. home, clinic, workplace	Page 8

15. Presence of non-participants	Was anyone else present besides the participants and researchers?	No
16. Description of sample	What are the important characteristics of the sample? e.g. demographic data, date	Pages 7 and 11
Data collection		
17. Interview guide	Were questions, prompts, guides provided by the authors? Was it pilot tested?	See additional files and Methods (pages 7 through 10)
18. Repeat interviews	Were repeat inter views carried out? If yes, how many?	No
19. Audio/visual recording	Did the research use audio or visual recording to collect the data?	Face-to-face interviews were audio recorded (page 10).
20. Field notes	Were field notes made during and/or after the inter view or focus group?	Yes
21. Duration	What was the duration of the inter views or focus group?	Semi-structured interviews with patients lasted on average 15 min; interviews with HCPs lasted on average 30 minutes; and interviews with hospital officials lasted on average 28 minutes (see Table 1, page 11).
22. Data saturation	Was data saturation discussed?	Yes. See Methods (page 8) and Limitations (page 29)
23. Transcripts returned	Were transcripts returned to participants for comment and/or correction?	No
**Domain 3: analysis and findings**		
Data analysis		
24. Number of data coders	How many data coders coded the data?	Two (page 12)
25. Description of the coding tree	Did authors provide a description of the coding tree?	No
26. Derivation of themes	Were themes identified in advance or derived from the data?	No
27. Software	What software, if applicable, was used to manage the data?	None
28. Participant checking	Did participants provide feedback on the findings?	No
Reporting		
29. Quotations presented	Were participant quotations presented to illustrate the themes/findings? Was each	Yes. See Results (pages 11-23)

	quotation identified? e.g. participant number	
30. Data and findings consistent	Was there consistency between the data presented and the findings?	Yes. See Discussion (pages 23-28)
31. Clarity of major themes	Were major themes clearly presented in the findings?	Yes. See Results (pages 11-23)
32. Clarity of minor themes	Is there a description of diverse cases or discussion of minor themes?	Yes. See Results (pages 11-23)
