# Supplementary material for: The views of patients, healthcare professionals and hospital officials on barriers to and facilitators of quality pain management in Ethiopian hospitals: A qualitative study
Source: PLoS One. 2019 Mar 14;14(3):e0213644. doi: 10.1371/journal.pone.0213644 (PMC6417681; doi:10.1371/journal.pone.0213644)
Supplement: S1 File — (PDF) [file pone.0213644.s003.pdf]

S1 File. Interview guide.

Topic guide for interviews with healthcare professionals
ለጤና ባለሙያዎች የተዘጋጀ የቃለምልልስ መምሪያ

1. Would you be kind enough to tell me what you do to manage pain in the surgical patient after surgery?
   እባክዎትን እሰኪ ከቀዶ ህክምና በኋላ በሚከሰተው የህመም ስሜት ለሚችገር ህመምተኛ ምን እያደረጉ ይገኛለ እስኪ እነደው ድርሻዎትን ቢያካፍሉኝ ?
   - Subsequent questions will be asked to clarify and further explore barriers influencing postoperative pain management.
     ከዚህ ጋር ተያይዞ ተከታታይ የሆኑ ጥያቆዎች ይጠየቃሉ። እነዚህ ጥያቆዎች ለህመም ስቃይ ህክምናው ማነቆዎችን ለመረዳት የታለሙ ናቸው።

2. Would you please share an example of a time when you worked to manage postoperative pain in a patient?
   እስኪ እባክዎትን ምሳሌ በመጥቀስ ከቀዶ ህክምና በኋላ ህመም ውስጥ የነበረን ህመምተኛ ያከሙበትን ሁኔታ ይገልጹልኛል
   - Was it successful or unsuccessful? How or why?'
     የተሳካ ነበረ ወይንስ አልተሳካም እንዴት ልምን

3. In your opinion, what are the barriers to proper management of pain in postoperative patients?
   እሰከ እነደው በእርስዎ አስተሳሰብ ከቀዶ ህክምና በኋላ የህመም ስሜትን ለመቆጣጠር እንቀፋት ይሆናሉ የሚሏቸውን ምክንያቶች ቢያስረዱኝ

4. In your opinion, what are the solutions to achieve adequate/satisfactory pain management? Probe questions will be asked, such as "What do you mean by that?" and "Can you elaborate more on that, please?" All interviews will be audio recorded and last between 15 and 20 min.
   የበለጠ ለመረዳት እናዲያመች ምን ማለትዎ ነው፣ እስኪ በደንብ ሊያብራሩልኝ ይችላሉ እና የመሳሰሉ የ ማነቃቂያ ጥያቄዎች ይጠየቃሉ።ሁሉም ቃለ ምልልሶች ከ 15-20 ደቂቃ ይቆያሉ እንዲሁም በድምጽ መቅረጫ ይቀዳሉ።

Guide for interviews with patients
ለህሙማን የተዘጋጀ መምሪያ

1. Tell me about your postoperative pain and pain relief experiences
   እባክዎትን ከቀዶ ህክምና በኋላ ስለነበርዎት የህመም ስሜት እና የ ህመም ማስተገሻ ቢነገሩኝ

2. Tell me about a specific pain situation: what happened? (How do they describe their pain?)

ለመረዳት እንዲያመች ስለነበርዎት አንድ አጋጣሚ (ከህመም ስሜቱ ጋር/ ከቁስሉ የህመም ስሜት) በተያያዘ ማለቴ ነው እስኪ ምን ሆነ ምን ተሠጥዎት

3. What is your perception of pain management? (In your opinion, is it important to treat it? How do you cope with it?)
   ከቀዶ ህክምና በኋላ ህመምን ለማከም የተለያዩ ዘዶዎችን መጠቀም እንዴት ነው በእርስዎ አስተሳሰብ ጥሩ ይመስልዎታል እርስዎ እንዴት ተቋሙት

4. What relieved or increased your pain?
   ምን አሻልዎት/ ምን አባሰብዎት

5. What was the barrier to pain treatment in your opinion? How was it a barrier?
   በእርስዎ እይታ እንቅፋት/ አዳጋች የሆነብዎት ምንድን ነው እንዴት

6. Was anything done to relieve your pain? Who offered you help with your pain?
   የህመም ስሜትዎን ለማስታገስ የተደረገልዎት ነገር አለ ማን ነው የረዳዎት

7. If there was an option other than drugs for your pain, like massage or acupuncture, would you be happy to use it?
   Probe questions will be asked, such as “What do you mean by that?” and “Can you elaborate more on that, please?”
   All interviews will be audio recorded and last between 15 and 20 min.
   ከህመም የማስተገሻ መድሃኒቶች ውጪ ፣ ማለትም የሚዋጡም ሆነ በመርፌ ከሚሰጡ ውጪ እንደመታሸት እና የደረቅ መርፌ ህክምና አማራጭ ቢቀርብልዎት ለመጠቀም ፍቀደኛ የሚሆ ይመስልዎታል
   የበለጠ ለመረዳት እናዲያመች ምን ማለትዎ ነው፣ እስኪ በደንብ ሊያብራሩልኝ ይችላሉ እና የመሳሰሉ የ ማነቃቂያ ጥያቄዎች ይጠየቃሉ፡፡ሁሉም ቃለ ምልልሶች ከ 15-20 ደቂቃ ይቆያሉ እንዲሁም በድምጽ መቅረጫ ይቀዳሉ፡፡

Guide for interviews with hospital officials
በስልጣን እና የሃላፊነት ድርሻ ላይ ላሉ የጤና ባለሙያዎች የተዘጋጀ መምሪያ

1. What is your position in the hospital/university or college? And your role?
   በሆስፒታሉ ወይንም በዮኒቨርሲቲው ያለዎት የሃላፊነት ቦታ ምን ይባላል

2. In your opinion, what is most important for surgical patients?
   ለቀዶ ህክምና በሽተኞች በእርስዎ እይታ በጣም አስፈላጊው ምንድን ነው

3. In your opinion, what is postoperative pain and do you think patients’ pain is managed on your ward/in your hospital?
   ከቀዶ ህክምና በኋላ የህመም ስቃይ ስሜትን እንዴት ይገልጹታል
   እርስዎ በሚሰሩበት በዚህ ሆስፒታል ወይንም ዋርደ ውስጥ በደንብ የሚታከም ይመስልዎታል

4. If yes….what are the strategies? Like guidelines? Protocols?
   አዎን ካሉ እስኪ የሚከተሉትን የትገበራ ሂደት ወይንም ዘዴ፣ እንዲሁም የሚጠቀሙትን መመሪያ እና ሳይንሳዊ ቀመር ቢያስረዱን

5. Some people say pain management is a luxury concern and we have a lot to do first rather than worrying about a patient’s pain after surgery. Do you agree or not? Give reasons.
   አንዳንዶች የቀዶ ህከምናው ነው እንጀ ዋናው ከዛ በኋላ የሚከሰተው የህመም ስሜት ቀላል እና ሊካበድ የማይገባው ነው፡፡ ሌሎች ልናደርጋቸው የሚገቡን ብዙ ነገሮች አለ እርሱ ብዙም አያሳስብም ይላሉ፡፡ እርስዎ በዚህ ሀሳብ ይስማማሉ ወይነስ አይስማሙም እስኪ ምክንያተዎን ዘርዘር አድርገው ያስረዱን

6. Do you continuously monitor how healthcare professionals manage pain after operations? If not, why? If yes, how?
   በሃላፊነት በታ ላይ እንደመሆንዎ፣ ባለሙያዎች ከ ቀዶ ህክምና በኋላ በትክክል ህመሙን መቆጣጠር ወይንም አለመቆጣተራቸውን በሚገባ እና በማያቋርጥ መልኩ ይከታተላሉ፡፡

7. Why do you think pain management **is** important? / Why do you think pain management **is not** important?
   ለምን ይመስልዎታል የህመም ህክምና ጠቃሚና አስፈላጊ ነው የሚባለው/ ለምን ይመስልዎታል የህመም ህክምና ጠቃሚና አስፈላጊ አይደለም የሚባለው/

8. In your opinion what is the best **strategy/ approach** to adequately manage postoperative pain?
   እንደው እንደረስዎ ከሆነ በተገቢው መልኩ የህመም ስቃይን ለመቆጣጠር እና ለመቀነስ ጥሩ የትግበራ ዘዴ ወይንም የሃሳብ ቀመር ምን ይመስልዎታል

9. Do you think all the necessary drugs/human resources for pain management are available? If not, why? If yes, can you give example?
   Probe questions will be asked, such as “What do you mean by that?” and “Can you elaborate more on that, please?” All interviews will be audio recorded and will last between 15 and 30 min.

   ሁሉም ግብአቶች ማለት የሰውም፣ የንብረትም፣ የመድሃኒትም ሆነ ሌሎች ይህን የህመም ስቃይ ለመቆጣጠር የተሟሉ ይመስልዎታል አልተሟሉም ካሉ ቢያብራሩልኝ
   የበለጠ ለመረዳት እናዲያመች ምን ማለትዎ ነው፣ እስኪ በደንብ ሊያብራሩልኝ ይችላሉ እና የመሳሰሉ የ ማነቃቂያ ጥያቄዎች ይጠየቃሉ፡፡ሁሉም ቃለ ምልልሶች ከ 15-30 ደቂቃ ይቆያሉ እንዲሁም በድምጽ መቅረጫ ይቀዳሉ፡፡
